# A Novel Mutation c.3392G>T of *COL2A1* Causes Spondyloepiphyseal Dysplasia Congenital by Affecting Pre-mRNA Splicing

**DOI:** 10.3389/fgene.2022.827560

**Published:** 2022-04-05

**Authors:** Lihong Fan, Longfei Ji, Yuqing Xu, Guosong Shen, Kefeng Tang, Zhi Li, Sisi Ye, Xueping Shen

**Affiliations:** ^1^ Center of Prenatal Diagnosis, Huzhou Maternity & Child Health Care Hospital, Huzhou, China; ^2^ Department of Clinical Laboratory, The First People’s Hospital of Huzhou, Huzhou, China; ^3^ Department of Reproductive Genetics, Women’s Hospital, School of Medicine Zhejiang University, Hangzhou, China

**Keywords:** spondyloepiphyseal dysplasia congenital, *COL2A1*, next-generation sequencing, minigene, in-frame deletion, prenatal diagnosis

## Abstract

Spondyloepiphyseal dysplasia congenital (SEDC) is a rare chondrodysplasia caused by dominant pathogenic variants in *COL2A1*. Here, we detected a novel variant c.3392G > T (NM_001844.4) of *COL2A1* in a Chinese family with SEDC by targeted next-generation sequencing. To confirm the pathogenicity of the variant, we generated an appropriate minigene construct based on HeLa and HEK293T cell lines. Splicing assay indicated that the mutated minigene led to aberrant splicing of *COL2A1* pre-mRNA and produced an alternatively spliced transcript with a skipping of partial exon 48, which generated a predicted in-frame deletion of 15 amino acids (p. Gly1131_Pro1145del) in the COL2A1 protein. Due to the pathogenicity of the variation, we performed prenatal diagnosis on the proband’s wife, which indicated that the fetus carried the same mutation.

## Introduction

Spondyloepiphyseal dysplasia congenital (SEDC) is a rare genetic disorder characterized by a short trunk, cervical spine subluxation, scoliosis, coxa vara, kyphosis, and metaphyseal changes (Xiong et al., 2018). Patients may also show extra-skeletal abnormalities such as myopia, hearing loss, and cleft palate (Deng et al., 2016; Zheng et al., 2020). The various mutations in the *COL2A1* gene, which contains 57 exons are the genetic cause of SEDC (Anderson et al., 1990; Nishimura et al., 2005).

Until today, according to HGMD (professional 2020.4), at least 130 different *COL2A1* mutations have been reported to be causally associated with SEDC. Most of these reported mutations were analyzed primarily at the genomic level (Dasa et al., 2019; Zheng et al., 2020). Only in a few studies have the effects of mutations been confirmed at both DNA and RNA levels (Bruni et al., 2021). A major concern is that we may detect many genomic DNA (gDNA) substitutions of unknown significance, and their pathogenicity is sometimes in urgent need of accurate assessment, especially when prenatal diagnosis and genetic counseling are required. A considerable number of studies have shown that exonic single-nucleotide variants can affect RNA splicing (Inoue et al., 2020; Wang et al., 2020). The most effective way to identify splicing alterations is to analyze the mRNA extracted from the patients’ relevant tissue. But in fact, this type of sample is not always available. In addition, it is often uneasy to analyze mRNA due to its instability and low expression level in peripheral leukocytes. Now minigene analysis has emerged as an alternative method to preliminarily assess whether a particular variant affects pre-mRNA splicing (Putscher et al., 2021).

In this study, we detected a novel variant in an SEDC family and further analyzed its pathogenicity by generating an appropriate minigene construct, followed by prenatal diagnosis and genetic counseling.

## Patients and Methods

### Case Presentation

**FIGURE 1:**
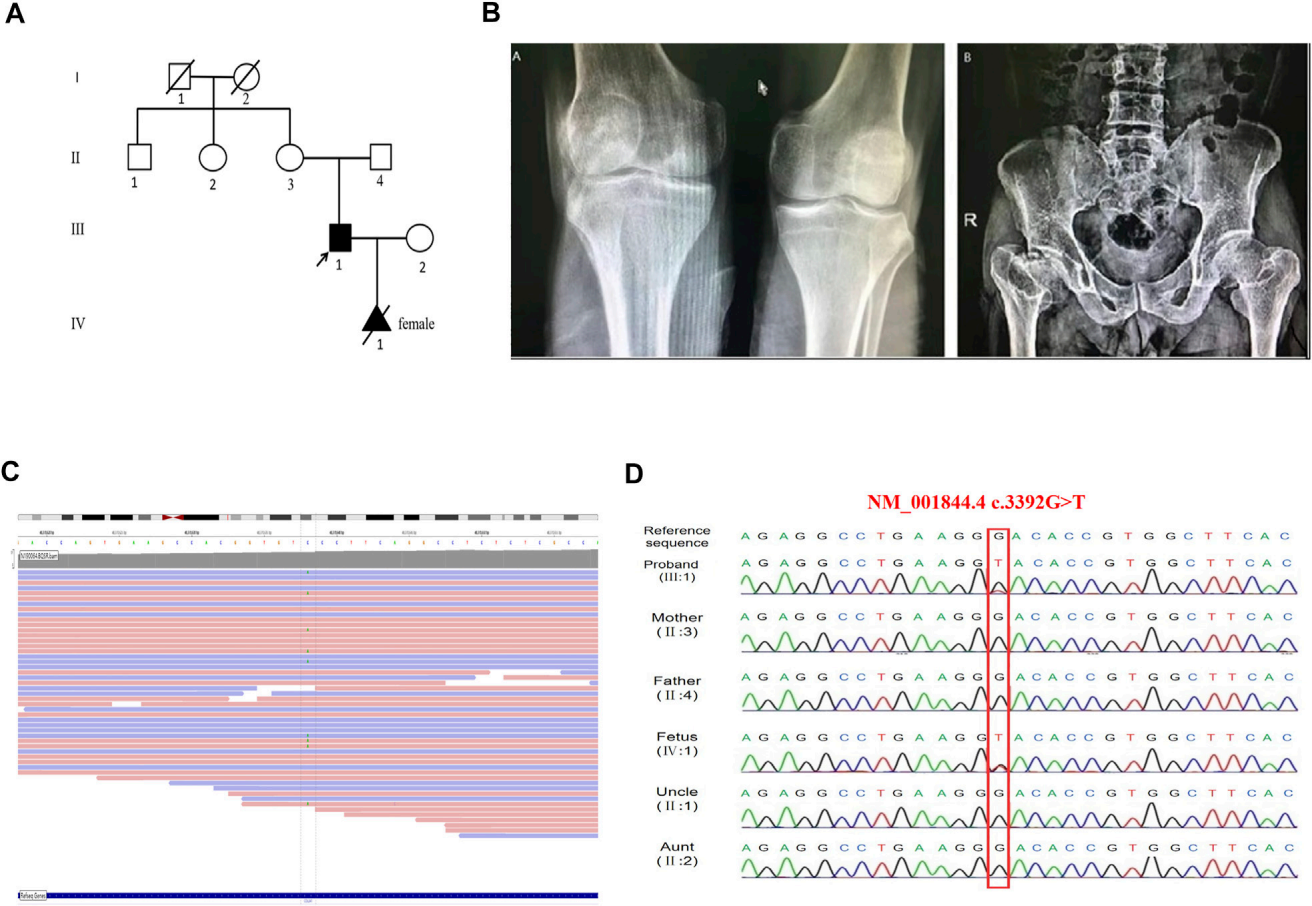
Feature of the proband and the *COL2A1* mutation in this family. **(A)** The pedigree of the proband. **(B)** Radiographs of the proband. The lower femur and the upper tibia are flattened and narrowed (left panel), the bilateral femoral heads are flattened, and the femoral neck is shortened (right panel). **(C)** Screenshot of the Integrative Genomics Viewer. Alternative alleles at *COL2A1* c.3392 position are marked. The total depth of sequencing coverage at this position was 44 reads. The allele frequency was 20% of tatal depth corresponding to the ref/alt was 35/9, suggesting mosaicism. **(D)** The Targeted next-generation sequencing and Sanger sequencing showed the proband and fetus carring the same mutation of c.3392G > T.

The proband (Ⅲ 1, Figure 1A) was a 36 years old man, with a height of 143 cm. He had an abnormal gait when he was 1 year old and then X-ray films showed hip dislocation (data not shown). Growth was markedly tardy from 13 years old and the pain in the hip appeared since the age of 25 years old, especially after long-distance walking. His eyesight, hearing, and intelligence were normal. X-ray films revealed flattened bilateral femoral heads, shortened femoral neck, flattened and narrowed articular surface of the lower femur and upper tibia (Figure 1B). His parents were healthy as neither of them displayed any signs of skeletal deformities or extra-skeletal deformities. As the proband’s wife was pregnant at the first visit, both the proband’s genetic diagnosis and prenatal diagnosis needed to be provided.

The current investigation was approved by the Ethics Committee of Huzhou Maternity & Child Health Care Hospital. All participants were provided their written informed consents.

### Targeted Next-Generation Sequencing and Data Analyses

A targeted next-generation sequencing panel was used to capture all exon sequences of 2,740 genes known to be associated with skeletal disorders. The peripheral blood sample of the proband was collected. Genomic DNA was then extracted using a Lab-Aid 820 DNA blood Mini Kit (Zeesan, China). Exome capture was performed using SureSelect Human All Exon V6 kit (Agilent Technologies, Santa Clara, CA, United States), followed by sequencing using an Illumina HiSeq2000 system (Illumina, San Diego, CA, United States). Whereafter, multiple database annotations (such as dbSNPs, ESP6500, GnomAD, 1,000 genomes, HGMD, LOVD 3.0, and ClinVar) were performed simultaneously to identify single nucleotide variants (SNVs). Finally, Sanger sequencing was performed to confirm the variant and evaluate the mode of inheritance.

### Splicing Prediction

To assess the presumptive effect of this variant on splicing, three different *in silico* prediction tools: SpliceAI (https://spliceailookup.broadinstitute.org/), Human Splicing Finder-Version 3.1 (HSF, http://www.umd.be/HSF3/HSF.shtml), and CBS were used.

### Minigene Splicing Assay

To create hybrid minigene constructs we used two vectors including pcMINI and pcDNA3.1, which we developed previously. Two wild-type fragments, one containing partial intro47 (169bp)-Exon48 (108bp)-partial intro48 (218bp), another containing Exon47 (54bp)-intro47 (182bp)-Exon48 (108bp)-intro48 (244bp)-Exon49 (54bp) were amplified by NEST-PCR. Then we introduced mutations by site-directed mutagenesis using PrimeStar mutagenesis basal kit (Takara Bio Inc.), according to the manufacturer’s instructions. The wild-type and mutated amplified products were purified by a gel extraction kit and then respectively cloned into the pcMINI and the pcDNA3.1 expression vector at the KpnI and BamHI restriction sites to generate four minigene constructs: pcMINI-COL2A1-wt/mut, and pcDNA3.1-COL2A1-wt/mut. All the primers are shown in Supplementary Table S1.

After plasmid amplification in DH5*α* competent cells and plasmid extraction (SIMGEN), the sequences and correct orientations of all the recombinant vectors were checked by Sanger sequencing (Macrogen, Madrid, Spain). Then the hybrid minigenes were transfected into HEK293T and HeLa cells using Lipofectamine^®^ 2000 (Thermo Fisher Scientific, Waltham, MA, CA). Total RNA was extracted from cells after 48h using the Trizol reagent (TaKaRa) according to the manufacturer’s instructions. Reverse transcription was performed with the Hifair^®^ II 1st Strand cDNA Synthesis SuperMix for qPCR (gDNA digester plus) kit (Yeasen, Shanghai China) according to the manufacturer’s instructions. The cDNA was amplified by PCR, then PCR products were analyzed by electrophoresis on a 1.5% agarose gel and Sanger sequencing (Macrogen, Madrid, Spain).

### Prenatal Diagnosis

Ultrasound-guided amniocentesis was performed at 19^+2^ weeks of gestation to obtain 30 ml of amniotic fluid, 15 ml for cell culture, a necessary step for conventional karyotype analysis, and another 15 ml for DNA extraction, which was performed for Sanger sequencing. The DNA extraction procedure is the same as described above.

## Results

### Identification and Bioinformatics Prediction of c.3392G > T in *COL2A1*

A novel variant c.3392G > T (NM_001844.4) in exon 48 of *COL2A1* was identified in the proband. A lower-than-expected number of reads supporting the variant suggested possible mosaicism (Figure 1C). This variant was not included in the normal population database (dbSNPs, ESP6500, GnomAD, and 1,000 genomes databases) or, as a pathogenic variant, in HGMD (professional 2020.2), LOVD 3.0, and ClinVar databases. Sanger sequencing of the mutation site observed in the proband confirmed the presence of the wild-type sequence in the unaffected parents (Figure 1D), thus identifying a *de novo* mutation in the proband. Functional predictions of the missense mutation by REVEL, Polyphen2, and ClinPred are shown in Table 1. *In silico* tools, HSF, SpliceAI, and CBS predicted that c.3392G > T may affect *COL2A1* RNA splicing by creating a new donor site. The splicing score is also shown in Table 1.

**TABLE 1:** *In silico* analysis of the *COL2A1* variant c.3392G > T.

	Software	Value	Effect		Software	Δ Score	Effect
Missense variant prediction	REVEL	0.980	+	Splicing prediction	SpliceAI	0.98	+
	ClinPred	0.999	+		CBS	0.93	+
	Polyphen2	0.999	+		HSF		+

### Splicing Analysis of *COL2A1* c.3392G > T in the Minigene

**FIGURE 2:**
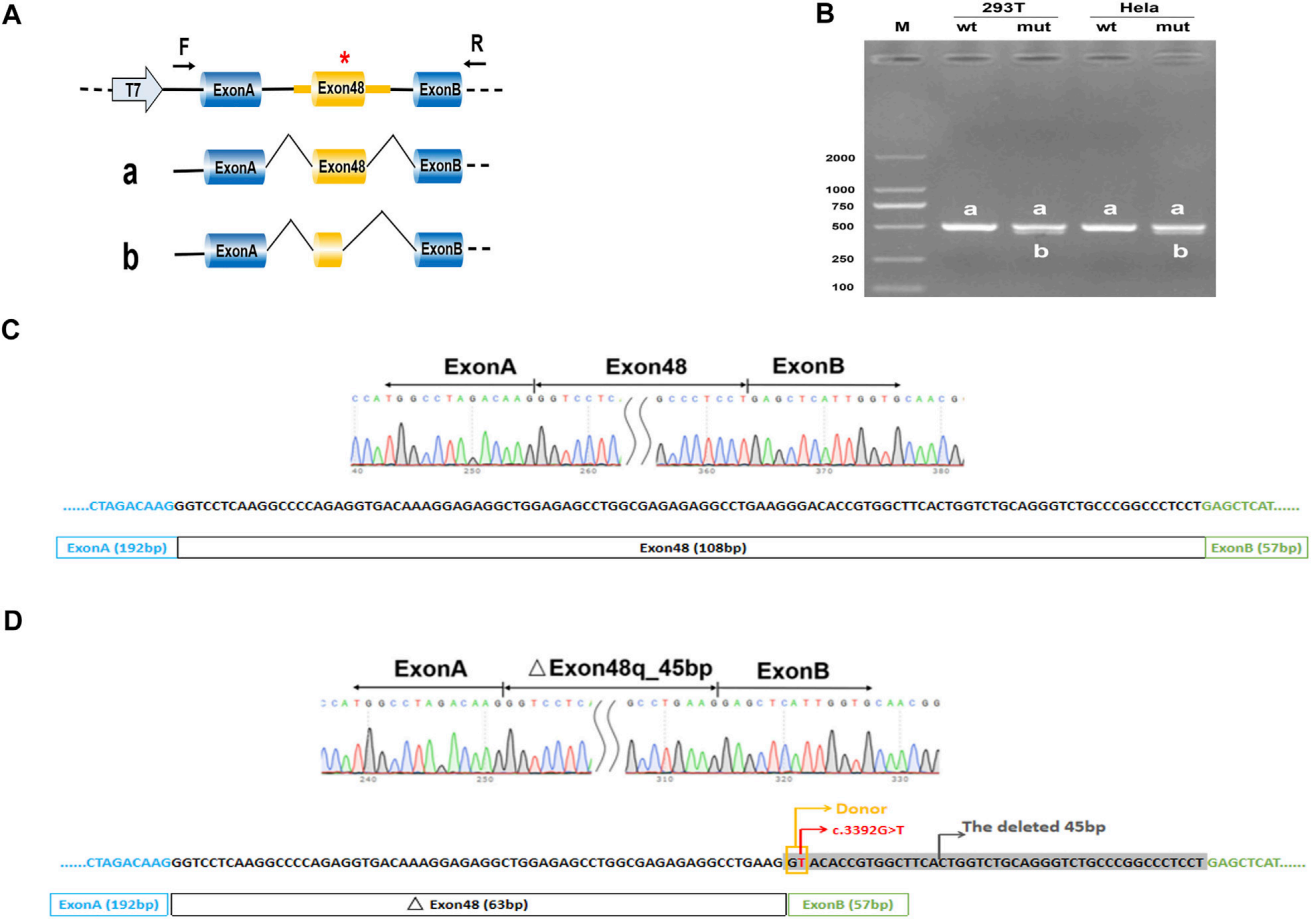
Minigene analysis based on pcDNA3.1-COL2A1-wt/mut recombinant vectors. **(A)**, Schematic illustration of cloned vectors. Red * indicates the mutation position. **(B)** Gel electrophoresis of the RT-PCR products in both 293T and HeLa cell lines. M means the DNA marker. Wild type (wt) exhibited a single band, a (full); mutant type (mut) exhibited double bands, a (full) and b (45 bp deletion). **(C)** Sanger sequencing result of the normal transcript. **(D)** Sanger sequencing result of the transcript with cryptic splicing caused by the mutation. The two bases in the yellow rectangle form a new donor, and the sequences in grey shade represent the missing 45 bases.

**FIGURE 3:**
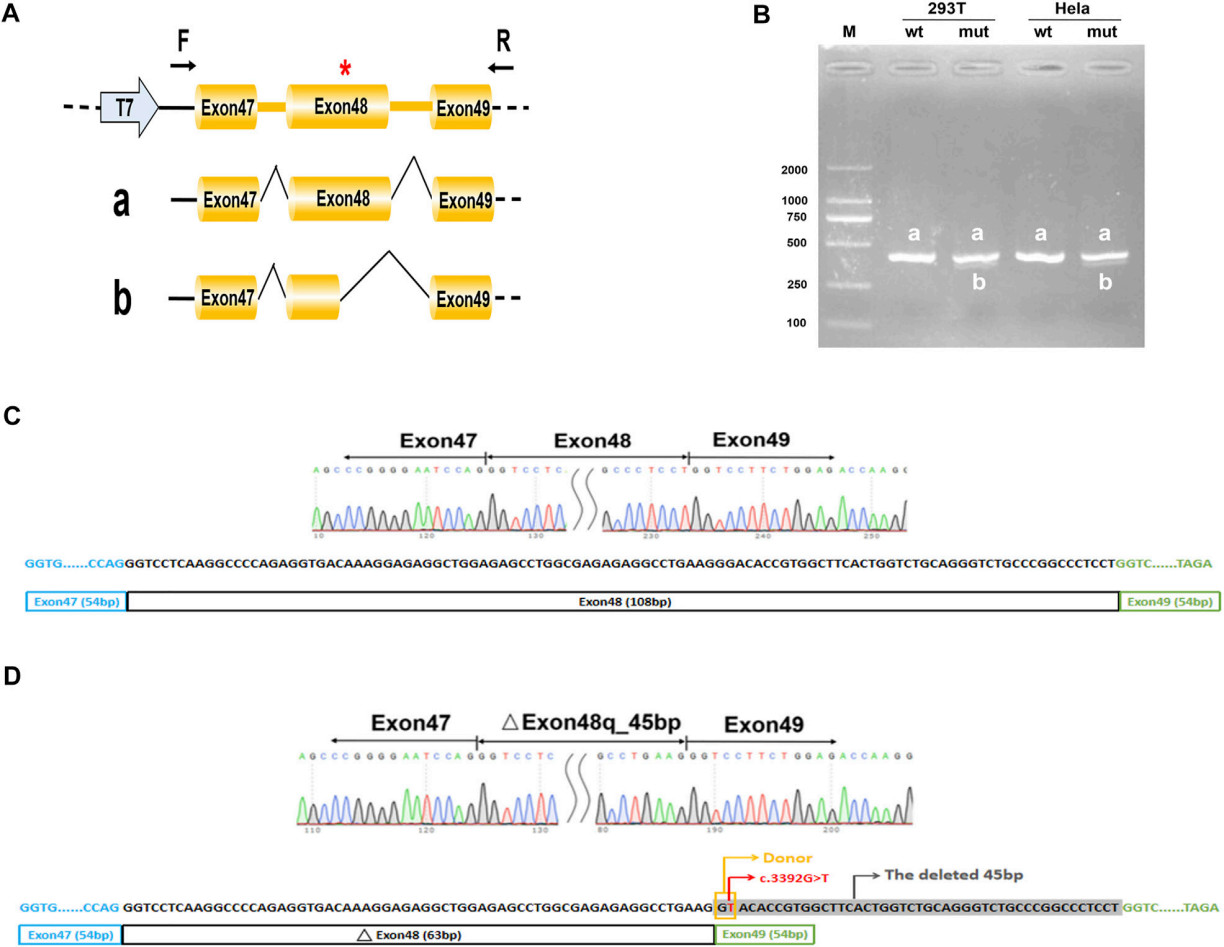
Minigene analysis based on pcMINI-COL2A1-wt/mut recombinant vectors. **(A)**, Schematic illustration of cloned vectors. Red * indicates the mutation position. **(B)** Gel electrophoresis of the RT-PCR products in both 293T and HeLa cell lines. M means the DNA marker. Wild type (wt) exhibited a single band, a (full); mutant type (mut) exhibited double bands, a (full) and b (45 bp deletion). **(C)** Sanger sequencing result of the normal transcript. **(D)** Sanger sequencing result of the transcript with cryptic splicing caused by the mutation. The two bases in the yellow rectangle form a new donor, and the sequences in grey shade represent the missing 45 bases.

Considering that the variant does not contain the splice acceptor site of exon 48 of *COL2A1*, we generated an appropriate minigene construct to explore the variant’s effect (Figure 2A, Figure 3A). By electrophoresis, the RT-PCR amplification products showed each mutant sample had two bands, one identical to the wild type and one slightly lower than the wild type (Figures 2B, 3B). Sanger sequencing of the long product showed that the wild type sequence came from the normal splicing, whereas the short product revealed a skipping of exon 48, which generated an in-frame deletion of 15 amino acids (p. Gly1131_Pro1145del) in the COL2A1 protein (Figures 2C,D, 3C,D).

**FIGURE 4:**
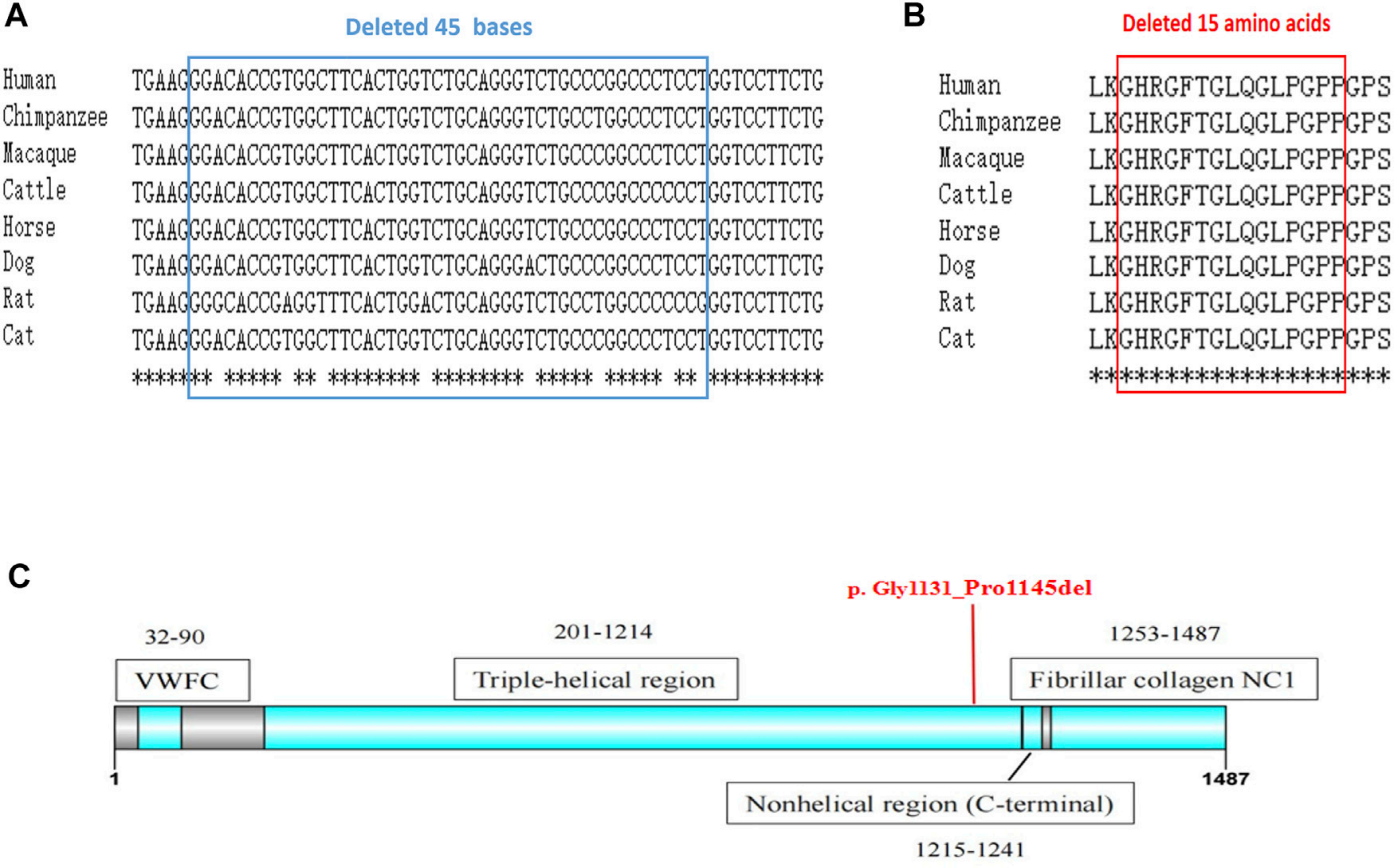
The conservation analysis and location of the mutation. **(A)** Alignment of *COL2A1* DNA sequences from multiple species. The affected bases affected by our novel mutation are highly conserved during evolution. **(B)** Alignment of COL2A1 amino-acid sequences from multiple species., analyzed by Online clustal omega (https://www.ebi.ac.uk/Tools/msa/clustalo/). **(C)** The deleted 15 amino acids locate in the triple helix region of COL2A1. The secondary structure of the protein is obtained from the uniprot database.

A comparison of the amino acid sequences indicates that these lost amino acids were highly evolutionary conserved among different species (Figures 4A,B). Moreover, the lost 15 amino acids locate in the triple helix region of COL2A1, predicating it may affect the protein spatial structure (Figure 4C). According to the American College of Medical Genetics and Genomics (ACMG) guidelines for interpretation of sequence variants (Richards et al., 2015), we consider this variant to be pathogenic (PS3+PM2_Supporting + PS4_Supporting + PP4+PS2).

### Prenatal Diagnosis and Genetic Counseling

**FIGURE 5:**
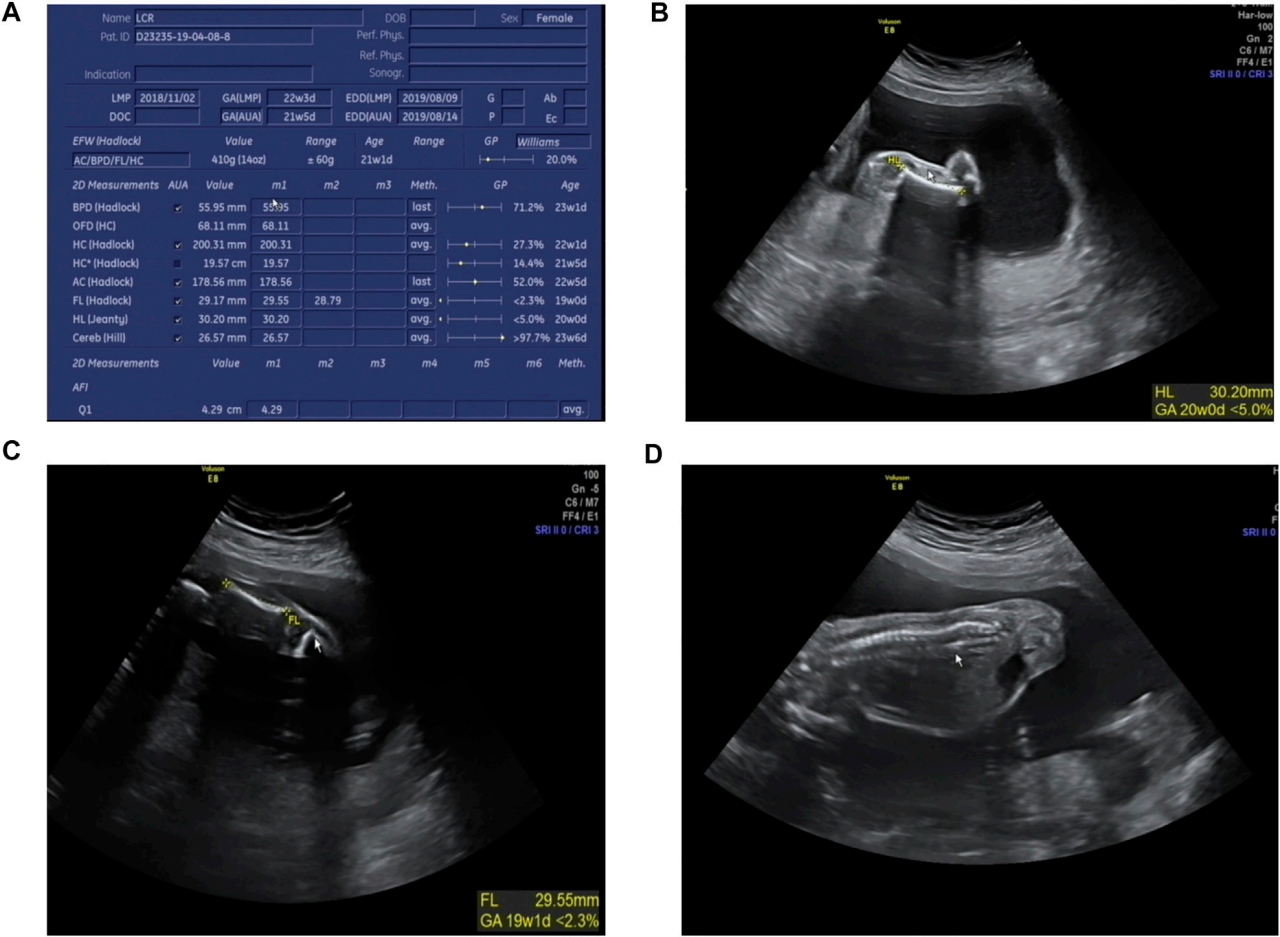
Radiographic features of the fetus (at 22^+3^ weeks’ gestation). **(A)** The fetus was generally smaller than the fetus at the same gestational age; **(B)** HL was equivalent to that of fetus at 20 weeks gestation; **(C)** FL was equivalent to that of fetus at 19^+1^ weeks’ gestation; **(D)** Fetus scoliosis could be seen.

The karyotype analysis revealed no abnormality. Sanger sequencing revealed that the fetus carried c.3392G > T in the heterozygous state (Figure 1D). The ultrasonography showed the fetus with corresponding structural malformations. At 22^+3^ weeks of gestation, head circumference, abdomen circumference, humerus length (HL), and femur length (FL) were at least lower than −2SD compared with the normal fetus at this gestational age (Figure 5). The couple chose to terminate the pregnancy due to molecular testing results and bone abnormalities.

## Discussion

SEDC is a heterogeneous group of skeletal dysplasias, associated with *COL2A1* mutations. Skeletal abnormalities in patients usually appear at birth and gradually progress (Dasa et al., 2019). Although therapeutic research has made progress in recent years, it is still incurable and may profoundly affect patients’ functional ability and quality of life. Prenatal diagnosis and genetic counseling are necessary for families with affected members.

Over the last decade, as gene sequencing has become widely used in clinical practice, thousands of rare variants have been identified. About 50% of these variants are missense variants, whose pathogenicity is generally difficult to assess accurately (Stenson et al., 2009). According to previous studies, approximately 15%–50% of exon single-nucleotide variations can affect protein function by affecting normal pre-mRNA splicing rather than substituting amino acids (Soukarieh et al., 2016; Baralle and Buratti, 2017). If only the DNA is examined, exonic variants far from the classical GT-AG splicing site might easily be misclassified as missense variants. Here, taking into account the patient’s typical clinical manifestations and that *in silico* splicing analysis showed the variant c.3392G > T in *COL2A1* had a high probability of disrupting gene splicing, we further conducted a minigene splicing assay. The results showed that the c.3392G > T variant generated a new donor and resulted in cryptic splicing, leading to an in-frame deletion of 15 amino acids in the triple-helical domain, which is considered the most important domain of COL2A1 (Zhang et al., 2020). According to the previous analysis of the mutation types and phenotypes, non-substitutions (deletions, duplications, delins, and insertions) are more likely to cause mild phenotypes (Zhang et al., 2020). The explanation given is that the non-substitutions may result in significantly abnormal amino acid sequences that are easily recognized and degraded, while the normal allele without variants provide the essential functions of type II collagen to sustain the basic function of cartilage tissue. On the other hand, the size of the in-frame deletion is considered to have a very significant correlation with the severity of the phenotype (Bruni et al., 2021). Variations with missing amino acids equal to or less than 18, which correspond to the number of residues in a turn of the extended left-handed helix formed by each collagen chain in the collagen triple helix, are associated with less severe phenotypes (Gelse et al., 2003). Deletions of more than 18 amino acid residues may form more extended loops, introducing larger structural defects in heterotrimers, while shorter loops might be more easily accommodated, producing fewer irregular protein assemblies (Bruni et al., 2021).

Mosaicism has been previously reported in at least seven families with *COL2A1*-related disease (Winterpacht et al., 1993; Spranger et al., 1994; Forzano et al., 2007; Désir et al., 2012; Nagendran et al., 2012; Yamamoto et al., 2020; Muirhead et al., 2021). In these studies, parents in a somatic mosaic status with the *COL2A1* mutation were clinically unaffected or showed a milder phenotype while offspring with heterozygous mutations tended to show a severe phenotype, the most severe cases even died neonatally (Forzano et al., 2007; Nagendran et al., 2012). Back to our study, the mutation c.3392G > T was detected in the proband at a low level although the sequencing result is not quantitative, and it has been proved as a loss of function variant, which is a common mechanism of *COL2A1*-related disorder. The features observed in the proband were slight, but fully consistent with SEDC, one type of *COL2A1*-related disorder. The fetus carried the heterozygous mutation and showed the key characteristics as early as 22^+3^ weeks. Consequently, we further clarified the genetic cause of SEDC in the family and speculated that the fetus would have more severe symptoms than the proband.

Before molecular defects in SEDC patients were reported, prenatal diagnosis of SEDC relied on ultrasound findings of shortened long bones, short femurs, a narrow chest, increased nuchal translucency, and so on (Patel and Filly, 1995; Chitty et al., 2006). According to previous studies, abnormal ultrasound findings of an SEDC fetus are not obvious until the second trimester of pregnancy. The earliest gestational age at which FL shortening could be detected is 16 weeks (Kirk and Comstock, 1990). In our study, the structural abnormalities of the fetus were first observed at 22^+3^ weeks gestation, which had exceeded the best period for prenatal diagnosis. Hence, for the families affected with SEDC, a prenatal molecular diagnosis should be completed as soon as possible. Obviously, confirmation of the proband’s pathogenic variation is a prerequisite for prenatal diagnosis and genetic counseling.

In conclusion, we verified the pathogenicity of the novel *COL2A1* variant c.3392G > T by next-generation sequencing and *in vitro* minigene assay, thus clarifying the cause of SEDC in the subject family and helping to guide for future pregnancy. In addition, our findings emphasize the importance of evaluating the impact of missense variants on pre-mRNA. The minigene assay may be a reliable and easy-to-use tool when a certain mutation is suspected to affect the normal splicing, and to collect the patient’s sample again for RNA transcription is impossible, or the mutant gene is not expressed or expressed too low in the blood.

## Data Availability

The datasets presented in this study can be found in online repositories. The name of the repository and accession number can be found below: SRA, NCBI; PRJNA817328.
