# Supplementary material for: A Novel Mutation c.3392G>T of COL2A1 Causes Spondyloepiphyseal Dysplasia Congenital by Affecting Pre-mRNA Splicing
Source: Front Genet. 2022 Apr 5;13:827560. doi: 10.3389/fgene.2022.827560 (PMC9174977; doi:10.3389/fgene.2022.827560)
Supplement: Supplementary file 1 [file Table1.DOCX]

Supplementary Table S1. Primer sequences used to amplify *COL2A1* genomic fragments

| **Process** | **Name of Prime** | **Primer sequence (5’→3’)** |
| --- | --- | --- |
| Step 1 of the NEST-PCR | 26625-COL2A1-F | GGCAGGAAGCAGCTCTAAGT |
|  | 28403-COL2A1-R | TGGTAGGGACACCTCGACAG |
| Step 2 of the NEST-PCR | 26897-COL2A1-F | TGATCGTGGTGAGACTGGTG |
|  | 28084-COL2A1-R | CAGGATAGCTCCATGGCTTG |
| Amplify the pcMINI-COL2A1-wt | pcMINI-COl2A1-KpnI-F | GGTAGGTACCTGTCCTGCACTGAGTCCCCA |
|  | pcMINI-COL2A1-BamHI-R | TAGTGGATCCCACGGTCAGCACAGACATAT |
| Amplify the left half of pcMINI mutant | pcMINI-COl2A1-KpnI-F | GGTAGGTACCTGTCCTGCACTGAGTCCCCA |
|  | COL2A1-mut-R | GTGAAGCCACGGTGTACCTTCAGGCCTCTCT |
| Amplify the right half of pcMINI mutant | COL2A1-mut-F | AGAGAGGCCTGAAGGTACACCGTGGCTTCAC |
|  | pcMINI-COL2A1-BamHI-R | TAGTGGATCCCACGGTCAGCACAGACATAT |
| Amplify the pcDNA3.1-COL2A1-wt | pcDNA3.1-COL2A1-KpnI-F | GCTTGGTACCATGGGTGCACAAGGCCCCATGGG |
|  | pcDNA3.1-COL2A1-BamHI-R | TAGTGGATCCTCTAGGGCCAGAAGGACCAG |
| Amplify the left half of pcDNA3.1 mutant | pcDNA3.1-COL2A1-KpnI-F | GCTTGGTACCATGGGTGCACAAGGCCCCATGGG |
|  | COL2A1-mut-R | GTGAAGCCACGGTGTACCTTCAGGCCTCTCT |
| Amplify the right half of pcDNA3.1 mutant | COL2A1-mut-F | AGAGAGGCCTGAAGGTACACCGTGGCTTCAC |
|  | pcDNA3.1-COL2A1-BamHI-R | TAGTGGATCCTCTAGGGCCAGAAGGACCAG |
